# In silico identification of substrate-binding sites in type-1A α-synuclein amyloids

**DOI:** 10.1016/j.bpj.2025.06.017

**Published:** 2025-06-18

**Authors:** Shraddha Parate, Fiamma Buratti, Leif A. Eriksson, Pernilla Wittung-Stafshede

**Affiliations:** 1 Department of Life Sciences, Chalmers University of Technology, Göteborg, Sweden; 2 Department of Chemistry and Molecular Biology, University of Gothenburg, Göteborg, Sweden; 3 Department of Chemistry, Rice University, Houston, Texas

## Abstract

Pathological amyloids associated with Parkinson and Alzheimer diseases have been shown to catalyze chemical reactions in vitro. To elucidate how small-molecule substrates interact with cross-β amyloid structures, we here employ computational approaches to investigate α-synuclein amyloid fibrils of the type-1A fold. Our initial binding pocket prediction analysis identified three distinct substrate-binding sites per protofilament, yielding a total of six sites in the dimeric type-1A amyloid structure. Molecular docking of the model phosphoester substrate para-nitrophenyl phosphate (pNPP), previously shown to be dephosphorylated by α-synuclein amyloids in vitro, was performed on the three identified sites. Docking was validated by molecular dynamics simulations for a period of 100 ns. The results revealed a pronounced preference for a single binding site (termed Site 2), as pNPP migrated to this region when primarily placed at the other two sites. Site 2 is located near the interface between the two protofilaments in a cavity enriched with lysine residues and histidine-50. Binding site analysis suggests stable, yet dynamic, interactions between pNPP and these residues in the α-synuclein amyloid fibril. Our work provides molecular-mechanistic details of the interaction between a small-molecule substrate and one α-synuclein amyloid polymorph. This framework may be extended to other reactive substrates and amyloid polymorphs.

## Significance

Pathological amyloids, traditionally viewed as chemically inert, have recently been shown to catalyze chemical reactions. This suggests a previously unrecognized chemical activity with potential implications for disease progression. We employed molecular docking and molecular dynamics simulations to elucidate the substrate-binding behavior of α-synuclein amyloids found in Parkinson disease. For a phosphoester substrate, we identified a preferential binding site at the protofilament interface in a lysine- and histidine-enriched cavity (here termed Site 2). The results provide a mechanistic basis for substrate recognition that may be extended to other substrates and amyloids. Further understanding of amyloid chemical catalysis may provide new approaches toward therapeutic targeting.

## Introduction

Amyloid fibrils are polymeric assemblies of protein monomers, connected by noncovalent interactions, with their β-strands oriented perpendicularly to the fibril axis in a cross-β structure (1). Numerous proteins can form amyloid fibrils under specific solvent conditions in vitro; however, their formation is predominantly associated with neurodegenerative disorders such as Alzheimer disease and Parkinson disease (PD) (1,2,3,4). In these disorders, amyloid fibrils are generally deemed as end products of aggregation, with intermediate species considered the most toxic to cells. Pathological effects of amyloid aggregation include mitochondrial dysfunction, impaired protein degradation, oxidative stress, and ultimately, cell death (5).

PD is the second most prevalent neurodegenerative disease and the most common movement disorder, with present treatment options limited to symptomatic relief (6,7). A hallmark of PD pathology is the presence of intraneuronal inclusions called Lewy bodies in patient brains, which primarily consist of amyloid fibrils formed by the protein α-synuclein (αSyn) (8,9,10). Genetic factors, including duplications, triplications, and point mutations in the αSyn gene, which enhance its expression and aggregation, are allied to familial cases of PD (11). Although soluble oligomeric forms of αSyn are purported to be the most toxic (12,13), αSyn amyloid fibrils themselves exhibit toxicity, with evidence indicating their ability to propagate between cells and cross the blood-brain barrier (14,15,16). Structurally, the ordered core of αSyn amyloids is hydrophobic and roughly comprises residues 50–94, although variations exist among different structures reported in the literature. The N-terminal region (residues 1–60) is amphipathic incorporating numerous basic residues, whereas the C-terminal region (residues 95–140) is acidic and comprises many negatively charged residues. Depending on conditions, mutations, and unknown factors, αSyn can adopt a range of amyloid folds (polymorphs), which were recently classified into different types and subtypes (17). Type-1A amyloids, exemplified by Protein Data Bank (PDB) structures 6H6B and 6A6B, are formed at physiological conditions (pH 7.0–7.5) by wild-type αSyn and are characterized by two protofilaments connected through a large interface comprising residues 50–57 in each monomer. Under acidic pH conditions or upon introduction of point mutations, other types of αSyn polymorphs can appear (17). In addition, αSyn amyloids from patient samples display yet other structures (18,19,20). It remains unclear how the different amyloid polymorphs connect to disease progression.

Previous studies have reported that amyloid fibrils, including those formed by amyloid-β (Aβ) in Alzheimer disease (21) and the glucose-regulating hormone glucagon (22), but not their monomeric counterparts, can catalyze pathological and metabolic reactions in vitro. These findings implied that amyloid fibrils, owing to their repetitive, in-register arrangement, expose distinct catalytic sites on their surfaces, enabling enzyme activity (23). In accordance with this, our group investigated whether αSyn amyloid fibrils also exhibit catalytic properties. Indeed, we discovered that at physiological conditions, wild-type αSyn amyloids, but not their monomeric counterparts, hydrolyzed ester and phosphoester bonds in model substrates (24,25). We also reported chemical alterations of a range of neuronal cell metabolites upon incubation with purified αSyn amyloids (26). More recently, we showed that at physiological conditions in vitro, αSyn amyloids can bind to and induce chemical damage in double-stranded DNA (27).

To gain deeper insight into the molecular basis of amyloid chemical reactivity, we aimed to identify substrate-binding sites on αSyn fibrils using computational approaches. For this, we leveraged molecular docking and molecular dynamics (MD) simulations to model the interaction of the phosphoester substrate para-nitrophenyl phosphate (pNPP) with the αSyn amyloid fiber surface. This model substrate was used in our previous in vitro work where we showed that wild-type αSyn amyloids catalyzed pNPP hydrolysis, but αSyn monomers and His50Ala-mutated αSyn amyloids did not (24). To facilitate comparison with the data from the in vitro experiments, we used high-resolution structures of αSyn amyloids with the type-1A fold, which is typically formed by wild-type αSyn at physiological conditions (17,24,25). The docking and simulation results taken together reveal that the preferred substrate-binding site (here termed Site 2) is found in a cavity near the interface between the two protofilaments that is enriched with lysine residues and histidine-50.

## Materials and methods

### Identification of potential binding sites

To investigate the presence of distinct binding sites on αSyn fibrils, we employed SiteMap, a computational module implemented within Schrödinger (28,29). SiteMap is designed to identify prospective substrate-binding sites in proteins. The algorithm evaluates interaction energies between the grid probes and the protein surface to identify energetically favorable binding spots (29). To characterize each binding region, SiteMap employs a series of physical descriptors, encompassing 1) the size of the site estimated by the number of site points, 2) the degree of enclosure by the protein, 3) the extent of exposure to solvent, 4) spatial tightness between the site points and the protein surface, 5) the hydrophilic and hydrophobic characteristics of the site including the balance between them, and 6) the extent with which a ligand can accept or donate hydrogen bonds (28).

### Protein preparation

We utilized the high-resolution structure of recombinant αSyn fibrils with PDB code 6H6B, which adopts a paired helical fibril conformation (30) classified as type-1A(17). In addition, the PDB structure 6A6B, which also represents a type-1A αSyn amyloid fold (31), was used to confirm the reliability of our findings. The retrieved protein structures were processed and refined employing the *Protein Preparation Wizard* tool in Maestro (Schrödinger 2024–4, www.schrodinger.com). During this process, bond orders were assigned, hydrogen bond networks were optimized, and the protonation states at physiological pH (pH 7.0) were determined using PROPKA (32). Subsequently, the optimized structures were subjected to restrained energy minimization utilizing the OPLS4 force field, with a root mean-square deviation (RMSD) convergence threshold of 3.0 Å for heavy atoms (33).

### Substrate preparation

The three-dimensional structure of pNPP was retrieved from the PubChem database (https://pubchem.ncbi.nlm.nih.gov/) and transferred into Maestro Schrödinger for further preparation (34). The substrate, pNPP, is a widely used phosphatase substrate that undergoes hydrolysis to release para-nitrophenol, a chromogenic product. The three-dimensional structure of pNPP was prepared employing the *LigPrep* module embedded in Schrödinger (Schrödinger 2024–4, www.schrodinger.com). The *Epik* machine learning program within *LigPrep* performs systematic conformational and ionization state generation while ensuring proper bond order assignments (35). Employing *Epik*, pNPP was processed by generating relevant protonation states at physiological pH (pH 7.0) and optimized with the OPLS4 force field to refine its geometry for subsequent docking and MD simulations (33,35).

### Molecular docking studies

Molecular docking of pNPP was performed at three distinct binding sites in 6H6B identified through SiteMap analysis. Receptor grids were generated based on the residues defining each binding site of the protein. Docking procedures were carried out employing the standard precision mode of Glide, which enables flexible ligand sampling, incorporating nitrogen inversions and ring conformation adjustments (36,37). Default parameters were applied, including a van der Waals scaling factor of 0.8 for nonpolar ligand atoms and partial charge cutoff of 0.15. The docking procedure included a postdocking minimization step, retaining up to 10 poses for pNPP at each binding site. For pNPP, the top-ranked docking pose was selected based on the Glide scoring function, ensuring an optimal assessment of substrate-receptor interactions with each binding site. Notably, in the Glide scoring framework, a lower (i.e., more negative) score indicates a stronger predicted binding affinity. The OPLS4 force field was used during the docking procedure (33).

### Binding pose metadynamics simulations

In this study, we also employed binding pose metadynamics (BPMD) simulations as implemented in Maestro Schrödinger to assess the stability of ligand binding in each of the identified binding pocket. Using BPMD, 10 independent simulations of 10 ns each are performed, utilizing the RMSD of the ligand heavy atoms from their initial conformation as a collective variable to guide simulations (38). The underlying principle of BPMD is that substrates exhibiting unstable binding with the receptor will undergo greater RMSD fluctuations under the influence of the biasing force, whereas stably bound ligands will maintain a more constrained binding pose. BPMD generates two key metrics to evaluate the substrate stability throughout the simulations. The PoseScore represents the average RMSD of the substrate relative to its initial binding pose, where a rapid increase in the PoseScore indicates that the substrate resides in an unstable energy minimum and may not have been accurately modeled. The PersistenceScore (or PersScore) quantifies the retention of hydrogen bond interactions between the substrate and the receptor over the course of the simulation. This score is estimated as the fraction of frames in the final 2 ns of the simulations that preserve the hydrogen bonding network of the initial complex, averaged across all 10 independent simulations. The PersScore ranges between 0 and 1, where a score of 0 indicates either an absence of initial substrate-receptor interactions or their complete loss during the simulation, and a score of 1 suggests that the substrate’s hydrogen bonding interactions remain fully preserved in the final 2 ns. Together, these metrics provide a robust evaluation of substrate stability and interaction persistence within the binding pocket.

### Molecular dynamics simulations

Classical MD simulations were performed for 100 ns to assess the stability of pNPP-αSyn complexes using the Desmond engine in Schrödinger (Schrödinger 2024-4, http://www.schrodinger.com) (39,40). Water molecules were modeled using the TIP3P force field, and periodic boundary conditions were employed with a 10-Å water buffer surrounding αSyn fibrils within an orthorhombic simulation box (41). To adjust the electroneutrality of the pNPP-αSyn complex systems, Na^+^ or Cl^−^ ions were added, maintaining a physiological salt concentration of 150 mM. The OPLS4 force field was used during all pNPP-αSyn complex simulations (33). All simulations were conducted under the isothermal-isobaric (NPT) ensemble, with temperature and pressure maintained at 300 K and 1.01325 bar atmospheric pressure using the Nosé-Hoover thermostat and Martyna-Tobias-Klein barostat with isotropic coupling, respectively (42,43,44). Postsimulation analysis for all systems, including the calculation of RMSD and protein-ligand contacts, was analyzed using the Simulation Interaction Diagram tool implemented within Schrödinger 2024-4 (http://www.schrodinger.com).

## Results

### Analysis of binding sites in amyloids

**Figure 1:**
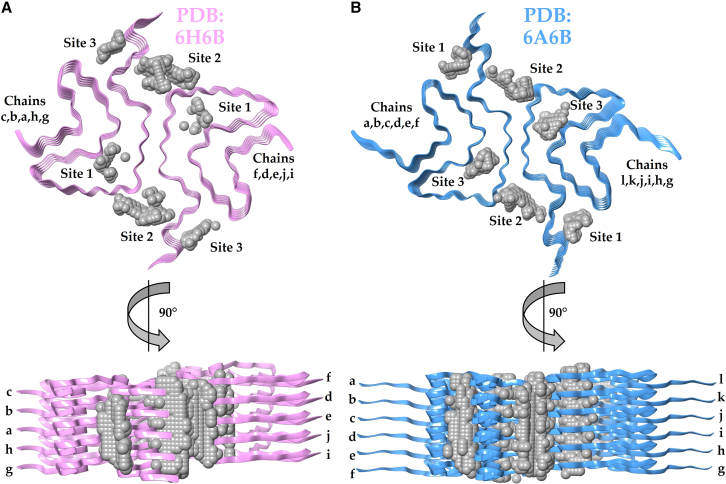
In silico identification of three distinct binding sites on (*A*) PDB: 6H6B (*in pink*) and (*B*) PDB: 6A6B (*in blue*) αSyn amyloid structures. The gray spheres represent residues predicted to contribute to potential substrate-binding pockets. Three such pockets were detected per amyloid protofilament when viewed from the top (*upper structures*), labeled as Sites 1, 2, and 3. Each site runs down the filament as each identical peptide layer is stacked in register on the next (*side views, lower structures*). Each amyloid fibril PDB structure consists of two protofilaments, with five peptide chains each, with distinct chain labels. In (*A*) PDB: 6H6B, the left protofilament comprises chains g, h, a, b, and c, with chain g being the outermost facing above, whereas the right protofilament contains chains i, j, e, d, and f, with chain i at the outermost layer above. In (*B*) PDB: 6A6B, the left protofilament includes chains a, b, c, d, e, and f with chain f as the outermost, and the right protofilament consists of chains l, k, j, i, h, and g, where chain g is outermost facing.

Using Schrödinger’s SiteMap, we identified three distinct binding sites denoted Sites 1–3 on the type-1A αSyn amyloids in PDB structures 6H6B and 6A6B (Fig. 1). Each protofilament harbors three binding sites, when viewed from the top, resulting in a total of six sites in the fibrillar assembly, as type-1A αSyn fibrils form a dimeric structure. Due to the repetitive packing of identical protein chains on top of each other in amyloids, the binding sites run along the long axis of the fibril (see side view, Fig. 1; the here used PDB structures contain five peptide layers each). Each binding site (defined from a top view) exhibited unique spatial and physicochemical properties based on SiteMap’s physical descriptors, including variations in size, degree of enclosure, solvent exposure, and binding potential. Site 1 is relatively buried within a cavity in the amyloid core and not easily accessible in a long amyloid fiber. This site includes residues Thr54, Val55, Ala56, Lys58, Glu61, Val63, Thr72, Gly73, Val74, and Thr75. Site 2 is positioned at a surface exposed cavity near the protofilament interface and encompasses residues Lys43, Lys45, and His50 from peptides in one protofilament and Glu57 and Thr59 from peptides in the other protofilament. Site 3, located in a cavity formed by the ordered N- and C-terminal parts of peptides within the same protofilament, comprises residues Val40, Gly41, Ser42, Thr44, Glu46, Lys80, and Val82.

### Interaction analysis through molecular docking, binding free energy, and binding pose metadynamics

**Figure 2:**
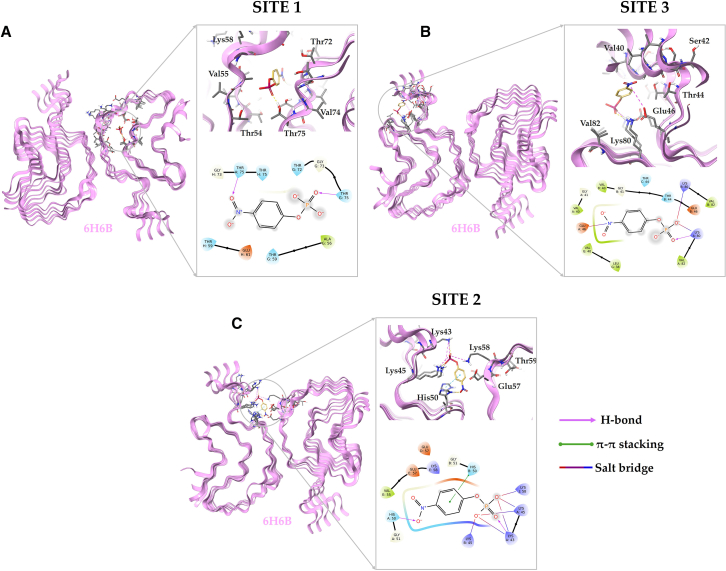
Molecular docking poses of pNPP when placed at the three identified binding sites (*A*, Site 1; *B*, Site 2; *C*, Site 3) on the type-1A αSyn amyloid structure 6H6B. In the zoomed-in three-dimensional interaction diagrams, key residues at each site are displayed along with hydrogen bonds and hydrophobic interactions formed with pNPP. The corresponding two-dimensional interaction maps offer a detailed overview of the molecular contacts between pNPP and residues at each binding site. Color-coded arrows indicate the types of interactions, including hydrogen bonds (*purple*), hydrophobic interactions (*green*), and electrostatic interactions (*red*), providing insight into the binding environment at each site. The two-dimensional and three-dimensional interaction diagrams were generated using Maestro Schrödinger.

Molecular docking of pNPP with the αSyn amyloid structures was performed at the three binding sites identified through SiteMap analysis, using the receptor grids defined by the vital residues at each site, as aforementioned. The docking scores for pNPP in each binding site in the amyloid structure 6H6B are summarized in Table 1. Corresponding data for the pNPP interactions with amyloid structure 6A6B can be found in Table S1. At Site 1, the pNPP molecule formed hydrogen bonds with Thr75 of chains A and G (chain labels defined in Fig. 1), highlighting key interactions within this buried binding pocket (Fig. 2 *A*). At Site 2, pNPP exhibited multiple interactions, forming hydrogen bonds with Lys43 of chain A and Lys45 of chains A and B. Additionally, His50 of chains A and B from one protofilament engaged in hydrogen bonding and π-π stacking interactions with pNPP, respectively (Fig. 2 *C*). Moreover, Lys58 of chain J from the opposing protofilament contributed to a salt bridge interaction. At Site 3, the pNPP molecule interacted with Lys80 of chains A and B via hydrogen bonding, while also forming a salt bridge with Glu46 of chain A (Fig. 2 *B*).

**Table 1:** Docking and Binding Pose Metadynamics (BPMD) Scores of pNPP at Three Distinct Binding Sites in αSyn Fibrils (PDB: 6H6B)

Binding Sites	Docking Scores (kcal/mol)	PerScores	PoseScores	MM/GBSA ΔG Bind (kcal/mol)
Site 1	−6.21	0.00	6.08	−7.49
Site 2	−6.87	0.29	5.99	−18.35
Site 3	−4.97	0.08	6.68	−1.95

The molecular docking and BPMD analysis of pNPP at the three identified binding sites of αSyn fibrils revealed distinct differences in binding stability and interaction persistence (Table 1). Among the identified sites, Site 2 exhibited the lowest docking score (−6.87 kcal/mol), indicating strong pNPP-αSyn interactions. Site 2 furthermore displayed the highest PersScore (0.29) among all sites, indicating that hydrogen bond interactions were partially retained throughout the BPMD simulations. PoseScore at Site 2 (5.99) is the lowest of all three sites, further supporting the structural stability of pNPP within this binding site. In contrast, Site 1 and Site 3 displayed lower stability, as evidenced by their higher PoseScores (6.08 and 6.68, respectively) and substantially lower PerScores (0.00 for Site 1 and 0.08 for Site 3), implying a loss of pNPP-αSyn interactions during BPMD. Although Site 1 exhibited a slightly better docking score (−6.21 kcal/mol) compared with Site 3 (−4.97 kcal/mol), its loss of hydrogen bond interactions (PersScore = 0.00) specifies a lack of stability in the bound state. This makes it unlikely to support sustained pNPP binding. Similarly, Site 3, although retaining some interactions (PersScore = 0.08), had the highest PoseScore (6.68), demonstrating greater fluctuations in the pNPP-αSyn conformation and a weaker pNPP stabilization.

In addition to the docking and BPMD scoring, the MM/GBSA binding free energy (ΔG bind) analysis further supports Site 2 as the most favorable binding site for pNPP. In this context, a lower (i.e., more negative) ΔG bind value indicates a stronger binding affinity. Site 2 exhibited the lowest ΔG bind value (−18.35 kcal/mol), indicating the strongest binding affinity in comparison to Site 1 (−7.49 kcal/mol) and Site 3 (−1.95 kcal/mol).

Overall, the docking, MM/GBSA, and BPMD results suggest that Site 2 is the most favored binding site for pNPP among the three identified sites. Site 2 demonstrates the strongest binding affinity and the highest degree of interaction persistence over the course of BPMD simulations.

### Analysis of substrate-amyloid contacts through MD simulations

**Figure 3:**
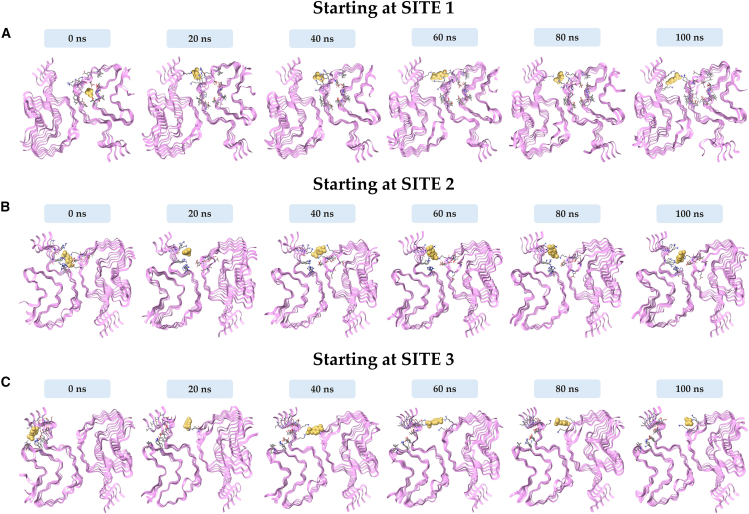
Binding profiles of pNPP at the three identified sites (*A*, Site 1; *B*, Site 2; *C*, Site 3) on αSyn fibrils (PDB: 6H6B) during different simulation time intervals over 100 ns. Snapshots show the position and behavior of pNPP relative to the three identified sites. Across all simulations, pNPP displayed a consistent tendency to migrate toward Site 2, regardless of its initial position, suggesting this site as the most likely binding site for catalysis.

**Figure 4:**
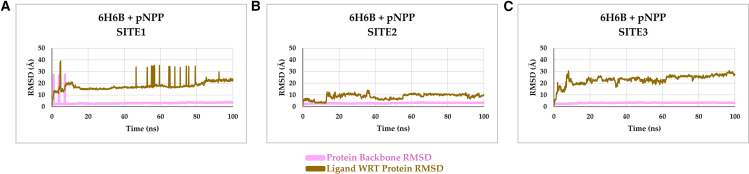
The backbone root mean-square deviation analysis of αSyn fibrils during MD simulation of 100 ns at the three identified binding sites. Substrate pNPP displays high fluctuations in relation to the protein backbone at Site 1 (*A*) and Site 3 (*C*), as compared with Site 2 (*B*). Root mean-square deviation profile of pNPP at Sites 1 and 3 shows sharp spikes, likely due to the compound transiently leaving and reentering the simulation box under periodic boundary conditions as it detaches from the binding cavity. In contrast, Site 2 (B) shows minimal fluctuations, indicating stable binding of pNPP throughout the simulation trajectory.

The stability and interaction of the different pNPP-αSyn complexes at three identified binding sites were next explored through 100 ns MD simulations. The data for amyloid structure 6H6B is described below, and the corresponding analysis for 6A6B is given in the supporting material (Figs. S1–S4). The MD simulations of pNPP at Site 1 of αSyn fibrils reveal significant displacement over time, ultimately leading to its relocation toward Site 2 (Fig. 3). At 0 ns, pNPP is initially positioned within Site 1 (docking pose), interacting with residues Thr75 through hydrogen bonding. However, as the simulation is initiated, pNPP exhibits increased mobility. Already by 20 ns, pNPP begins to shift away from Site 1 to instead adopt a more stable position within Site 2, engaging in interactions with residues Lys43 and Lys45. The transition of pNPP from Site 1 to Site 2 indicates that Site 1 does not provide a stable environment for substrate binding. The lack of stability of pNPP in Site 1 was also visualized using RMSD of the pNPP in relation to the protein backbone over 100 ns (Fig. 4). The backbone RMSD of αSyn remained consistently low throughout the simulation. In contrast, pNPP’s RMSD demonstrated significant fluctuations (Fig. 4 *A*) in line with relocation on the amyloid.

The MD simulation analysis of pNPP at Site 3 of αSyn fibrils also displays significant instability of pNPP similarly to when placed in Site 1 and leads to eventual migration of pNPP toward Site 2 (Fig. 3). Initially, at 0 ns (docking pose), pNPP is positioned within Site 3, interacting with Lys80 and Glu46. However, by 20 ns, pNPP begins to lose its interactions, drifting away from Site 3. As the simulation proceeds (40–60 ns), pNPP exhibits a high degree of mobility, failing to establish sustained interactions within Site 3. By the end of the simulation, pNPP is entirely stabilized within Site 2. These results suggest that Site 3 does not provide a suitable environment for substrate binding, as is also evident from the RMSD analysis (Fig. 4 *C*), which shows large fluctuations in accord with relocation.

To reveal the transition mechanism of pNPP from Sites 1 and 3 to Site 2, we analyzed the early frames of the MD trajectory. When pNPP was initially placed at Site 1, its relocation toward Site 2 began as early as frame 6 (0.5 ns). At this point, Lys58 engages in an electrostatic interaction with pNPP, effectively pulling it out of the Site 1 cavity. As pNPP migrates out of Site 1, it transiently interacts with Glu61, which appears to guide it further along the protein surface. By frame 75 (7.4 ns), pNPP becomes stably positioned within the Site 2 cavity, forming interactions with Lys43 in addition to Lys58. Over the remainder of the simulation, Lys45 also engages the substrate, contributing to the stabilization of pNPP at Site 2.

In the case of Site 3, pNPP initially attempts to leave the cavity through the terminal end, but this movement is hindered by transient tethering to Val40. Subsequently, pNPP alters its course and moves along the protein surface toward Site 2. At frame 73 (7.2 ns), Lys45 interacts with pNPP and facilitates its entry into Site 2. This interaction is further stabilized at frame 84 (8.3 ns), where pNPP engages Lys43. During the remainder of the simulation, additional interactions with Lys58 and Lys60 contribute to long-term stabilization of pNPP within the Site 2 pocket. These findings suggest that pNPP relocates by gradually swimming along the exterior of the fibril, rather than passing through the protofilament core. The transition of pNPP to Site 2 from both Sites 1 and 3 implies it is the most favored site for substrate interaction. To assess this conclusion, the substrate was next placed directly in Site 2.

Site 2 is characterized by residues Lys43, Lys45, His50, Glu57, and Thr59, located near the interface of the protofilaments. As stated above, the docking studies yielded favorable binding scores for pNPP at Site 2 of αSyn fibrils, suggesting a strong initial affinity. Subsequent MD simulations over a period of 100 ns showed that pNPP maintains stable interactions within this site throughout (Fig. 3). This is also evident from the low and stable RMSD found for pNPP in Site 2 throughout the trajectory (Fig. 4 *B*). Notably, during the simulation, pNPP forms persistent hydrogen bonds with Lys43 and Lys45 of chain A, as well as interactions with His50 of chains A and B.

Similar results were obtained from docking and simulation of pNPP with the αSyn amyloid structure 6A6B (Table S1; Figs. S1–S4). The docking and MM/GBSA (ΔG bind) scores for the 6A6B structure further supports Site 2 as the most favorable binding site for pNPP (Table S1). MD simulations after docking showed substrate migration from Site 3 to Site 2 and retention of pNPP in Site 2 when initially placed there (Fig. S2). However, over the course of 100-ns MD simulations, pNPP did not translocate from Site 1 to Site 2 in the 6A6B fibril structure (Fig. S2), as observed in the 6H6B structure. To explain the Site 1 discrepancy, we superimposed the 6H6B and 6A6B fibril structures (Fig. S5). Although the structures are very similar, Lys58 in 6A6B is directed toward Site 1 but toward Site 2 in 6H6B. This alternate Lys58 positioning may contribute to the stabilization of pNPP within Site 1 in the 6A6B fibril. Physiologically relevant amyloid fibrils typically comprise a much greater number of layers (many thousands) than the five-layered protofilaments used here. Given that the only plausible route of entry to Site 1 would be from the fibril ends, substrate accessibility to Site 1 in a full-length fibril in vivo will be significantly limited.

### Interaction profile of simulation complexes

**Figure 5:**
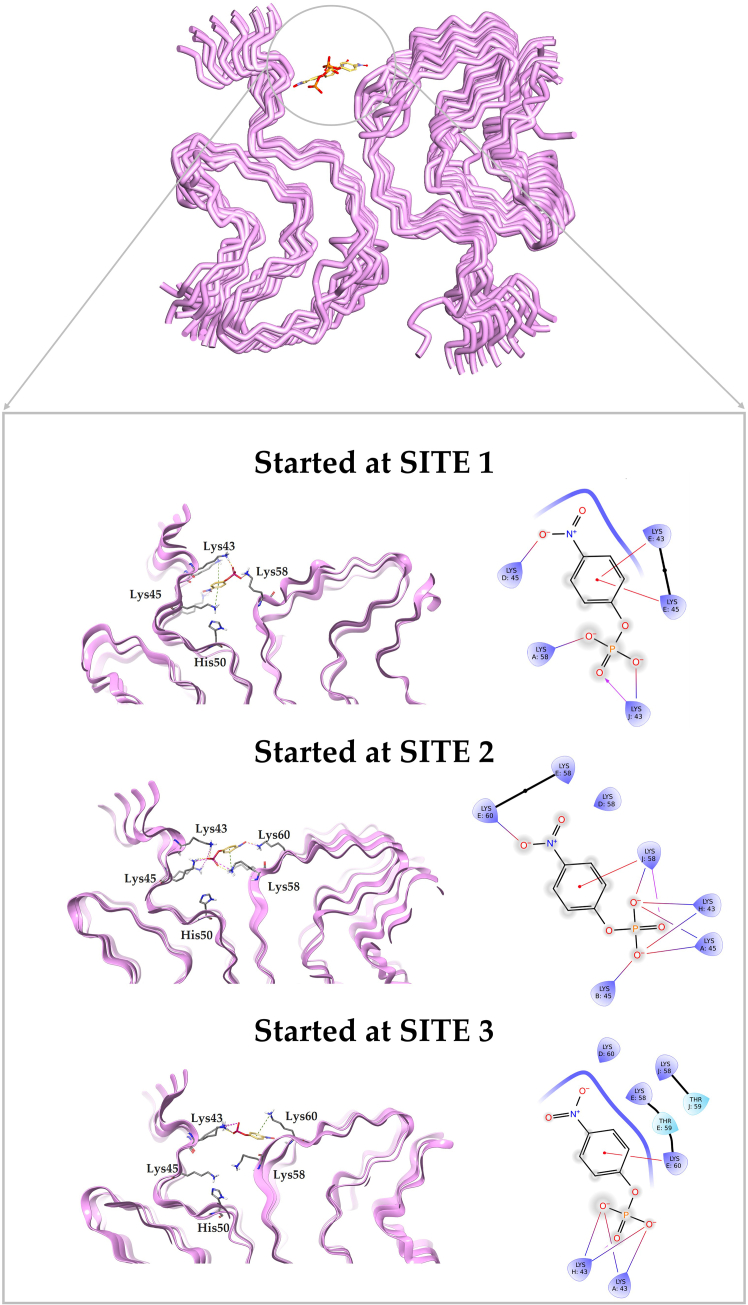
Representative snapshots after 100-ns simulations of 6H6B with pNPP, showing the preferential location at Site 2. The corresponding two-dimensional and three-dimensional interaction diagrams were generated using Maestro Schrödinger. See also Video S1. pNPP dynamics at Site 1 from 80 to 100 ns of MD simulation, Video S2. pNPP dynamics at Site 2 from 80 to 100 ns of MD simulation, Video S3. pNPP dynamics at Site 3 from 80 to 100 ns of MD simulation in the Supporting Material for illustration of the dynamics within the cavity.

To assess how pNPP interacts in the Site 2 pocket, we compared the three binding modes that were detected after 100-ns simulation when starting from pNPP docked in each of the three sites. The final sites in each simulation share a network of hydrogen bond donors and acceptors. A vital factor appears to be the positioning of hydrophilic residues, particularly Lys43 and Lys45 (Fig. 5). When docked at Site 1, pNPP migrated and stabilized at a position formed by Lys43, Lys45, and Lys58 interactions in Site 2 within 100 ns of MD. Similarly, starting from Site 3, pNPP relocated to a position involving Lys43, Thr59, and Lys60 in Site 2 during MD. When pNPP was placed at Site 2 at the start, it remained stably bound with a network of interactions involving Lys43, Lys45 in one protofilament and Lys58 and Lys60 of the adjacent protofilament throughout the simulation (Fig. 5). Across all final binding poses, positively charged lysine residues were prominently involved in anchoring the negatively charged phosphate moiety of pNPP through electrostatic interactions and hydrogen bonding, thereby contributing to its retention in the cavity.

The shared characteristics of the final binding sites across simulations (including the analogous analysis of 6A6B-pNPP binding modes in Site 2, Fig. S4) reinforce the idea that specific sidechain interactions, hydrogen bonds, and structural constraints play a crucial role in defining substrate-binding sites on αSyn fibrils. Nonetheless, it is evident that the interactions between pNPP and αSyn amyloids are dynamic within the Site 2 cavity (Videos S1, S2, and S3; morphs of last 20 ns of MD simulations).

Video S1. pNPP dynamics at Site 1 from 80 to 100 ns of MD simulation 200 frames shown with recording interval of 100 ps.

Video S2. pNPP dynamics at Site 2 from 80 to 100 ns of MD simulation 200 frames shown with recording interval of 100 ps.

Video S3. pNPP dynamics at Site 3 from 80 to 100 ns of MD simulation 200 frames shown with recording interval of 100 ps.

## Discussion

Although many αSyn studies focus on inhibitory effects of small molecules on amyloid formation, several small molecules have been identified to bind to αSyn amyloid fibers (45,46,47). With the increasing number of high-resolution cryo-EM studies of αSyn amyloid structures, and such structures of other protein amyloids, it is clear that each amyloidogenic protein may adopt a range of amyloid folds that, to date, differ between patient material and test tube experiments. It has been speculated that environmental conditions, posttranslational modifications, protein truncations, small-molecule interactions, other proteins, etc., may be responsible for the discrepancy, but it remains unknown. In addition to many αSyn amyloid folds, there are now also several high-resolution structures of αSyn amyloids that include ligands bound to specific sites (see https://people.mbi.ucla.edu/sawaya/amyloidatlas/).

We recently demonstrated that αSyn amyloids not only bind small molecules, but they can also do chemistry on such molecules (24,25). So far, we have found that αSyn amyloids can catalyze dephosphorylation and ester hydrolysis reactions in vitro. Using pNPP as a model phosphoester substrate, we showed that wild-type αSyn amyloids catalyzed pNPP hydrolysis, but αSyn monomers and His50Ala-mutated αSyn amyloids did not. To identify the molecular mechanism behind amyloid-mediated pNPP hydrolysis, we herein took a computational approach focusing on αSyn amyloids with the type-1A fold. This polymorph was selected because it is typically formed by wild-type αSyn at our experimental conditions (24,25).

Using computations, we unraveled that type-1A αSyn amyloids contain three distinct binding sites per protofilament (when viewed from the top; sites run along the amyloid long axis due to the repetitive nature of peptide packing), here labeled Sites 1 to 3. Site 1 is biologically irrelevant as it is within an enclosed cavity in the core of the amyloid. In contrast, Sites 2 and 3 are found on cavities on the amyloid surface and were recently shown to be involved in interactions with a range of chemical compounds (48). In that screening study, molecules such as classic dyes, imaging tracers, and more were tested for αSyn amyloid interactions using cryo-EM analysis resolving both binding sites and amyloid folds (48). Differential binding preferences to the type-1A αSyn amyloids were reported among the different chemical scaffolds tested, and many of the ligands harbored multiple binding sites. For example, Thioflavin-T, the common amyloid-staining dye, preferred the site we here labeled as Site 3, but it was also found in Site 2 in a fraction of the αSyn amyloids (48).

Coming back to our computational work, pNPP was found to favor Site 2 in the type-1A αSyn amyloids. Even when pNPP was docked in Site 1 or Site 3, it relocated to Site 2 in less than 20 ns of MD. Site 2 appears to facilitate stable binding through a network of hydrogen bonds and hydrophobic interactions, preventing pNPP dissociation over the course of at least 100 ns. The lysine residues (Lys43, Lys45, Lys60) likely play a crucial role in orienting and stabilizing the phosphate group of pNPP. His50, the sole histidine in the αSyn polypeptide, is also near the pNPP molecule in Site 2, likely contributing to the extended interaction network, but it does not make direct contacts throughout the simulations. The Site 2 properties found here align with experiments on designed synthetic amyloids that demonstrate the key role of polar residues for reactivity (49,50). For example, synthetic peptide-based amyloids with imidazole (histidine-like) and guanidinium (arginine-like) functional groups were shown to bind and hydrolyze phosphoester substrates (49).

Even if the type-1A αSyn polymorph has not yet been observed in patients with synucleinopathy diseases, it is commonly detected when aggregating wild-type αSyn at physiological conditions in vitro. We believe the general principles discovered here may be extended to small-molecule interaction and chemical reactivities of other, more disease-relevant, αSyn amyloid polymorphs. A recent in silico study proposed that the small-molecule polyphosphate (polyP) explained the “mystery density” observed in patient-derived αSyn fibrils (51). Using docking and MD simulations, along with in vitro binding studies with mutated αSyn amyloids, Lys43 and Lys45 were suggested to form the primary interface for the polyP interaction. These residues appeared to form a hydrogen bond network that stabilized polyP through salt bridges and electrostatic interactions (51). The common involvement of Lys43 and Lys45 in binding of both polyP and pNPP (in two different αSyn amyloid polymorphs) implies them as a general phosphate-binding “hotspot” in αSyn amyloids.

Electrostatic complementarity, along with structural constraints, may rationalize preferential binding of phosphate-containing substrates in specific cavities on amyloid surfaces. Even though the binding of pNPP is dynamic on a local level (see Videos S1, S2, and S3), its retention in a restricted cavity on the amyloid surface (here Site 2), which is somewhat shielded from water and exposes functional groups that make favorable interactions, may be sufficient to facilitate chemical bond cleavage. For biological relevance, and role in disease progression, further in vitro and in silico ligand-interaction studies are needed on αSyn amyloid polymorphs found in vivo. In addition, computations involving more than one ligand per amyloid structure (here, we used one ligand to one five-layered amyloid structure) may expose cooperativity in ligand binding along the cavities running down the amyloid long axis. Finally, it is important to address possible roles of the “fuzzy coat” (i.e., the floppy N- and C-termini of the αSyn peptides that protrude from the ordered amyloid core) in amyloid catalytic activity. These peptides are disordered and thus not captured by high-resolution structures. Still, their presence may promote (help capture substrates from surroundings) as well as hinder (block core access for larger substrates) catalytic activity depending on each substrate’s chemical and physical properties.

## Acknowledgments

The 10.13039/501100004359Swedish Research Council, 10.13039/501100004063Knut and Alice Wallenberg Foundation, 10.13039/100018880Sven and Lilly Lawski Foundation, and the 10.13039/100012538Swedish Cancer Foundation are acknowledged for funding.

## Author contributions

P.W.-S. and S.P. conceived the idea. P.W.-S., L.A.E., and S.P. designed experiments. S.P. performed experiments. P.W.-S., S.P., and F.B. analyzed data. P.W.-S. and S.P. wrote the manuscript. All authors edited the manuscript.

## Supporting material

Document S1. Figures S1–S5 and Table S1

Document S2. Article plus Supporting Material
