# Supplementary material for: In silico identification of substrate-binding sites in type-1A α-synuclein amyloids
Source: Biophys J. 2025 Jun 18;124(15):2418–27. doi: 10.1016/j.bpj.2025.06.017 (PMC12414660; doi:10.1016/j.bpj.2025.06.017)
Supplement: Document S1. Figures S1–S5 and Table S1 [file mmc1.pdf]

Biophysical Journal, Volume *124*

Supplemental information

In silico identification of substrate-binding sites in type-1A α-synuclein amyloids

Shraddha Parate, Fiamma Buratti, Leif A. Eriksson, and Pernilla Wittung-Stafshede

SUPPORTING MATERIAL

for

In silico Identification of Substrate Binding Sites in Type-1A α-Synuclein Amyloids

by Parate[1], Buratti[1], Eriksson[2], and Wittung-Stafshede[1,3]

[1]*Department of Life Sciences, Chalmers University of Technology, 412 96 Göteborg, Sweden*

[2]*Department of Chemistry and Molecular Biology, University of Gothenburg, 405 30 Göteborg, Sweden*

[3]*Department of Chemistry, Rice University, Houston, 77005 Texas, USA*

CONTENT:

This pdf

Table S1

Figures S1-S5

In separate files

Movies 1-3 showing pNPP dynamics at Site 1, 2 and 3, respectively, from 80–100 ns of MD simulation. In each case, 200 frames shown with recording interval of 100 ps.

Table S1.

Docking and binding pose metadynamics (BPMD) scores of pNPP at three distinct binding sites in type-1A αSyn fibrils PDB: 6A6B.

**Binding Sites**	**Docking Scores (kcal/mol)**	**PerScores**	**PoseScores**	**MM/GBSA ΔG Bind (kcal/mol)**
Site 1	-5.70	0.84	1.91	-9.63
Site 2	-5.98	0.27	8.33	-14.47
Site 3	-5.45	0.00	20.93	-8.00

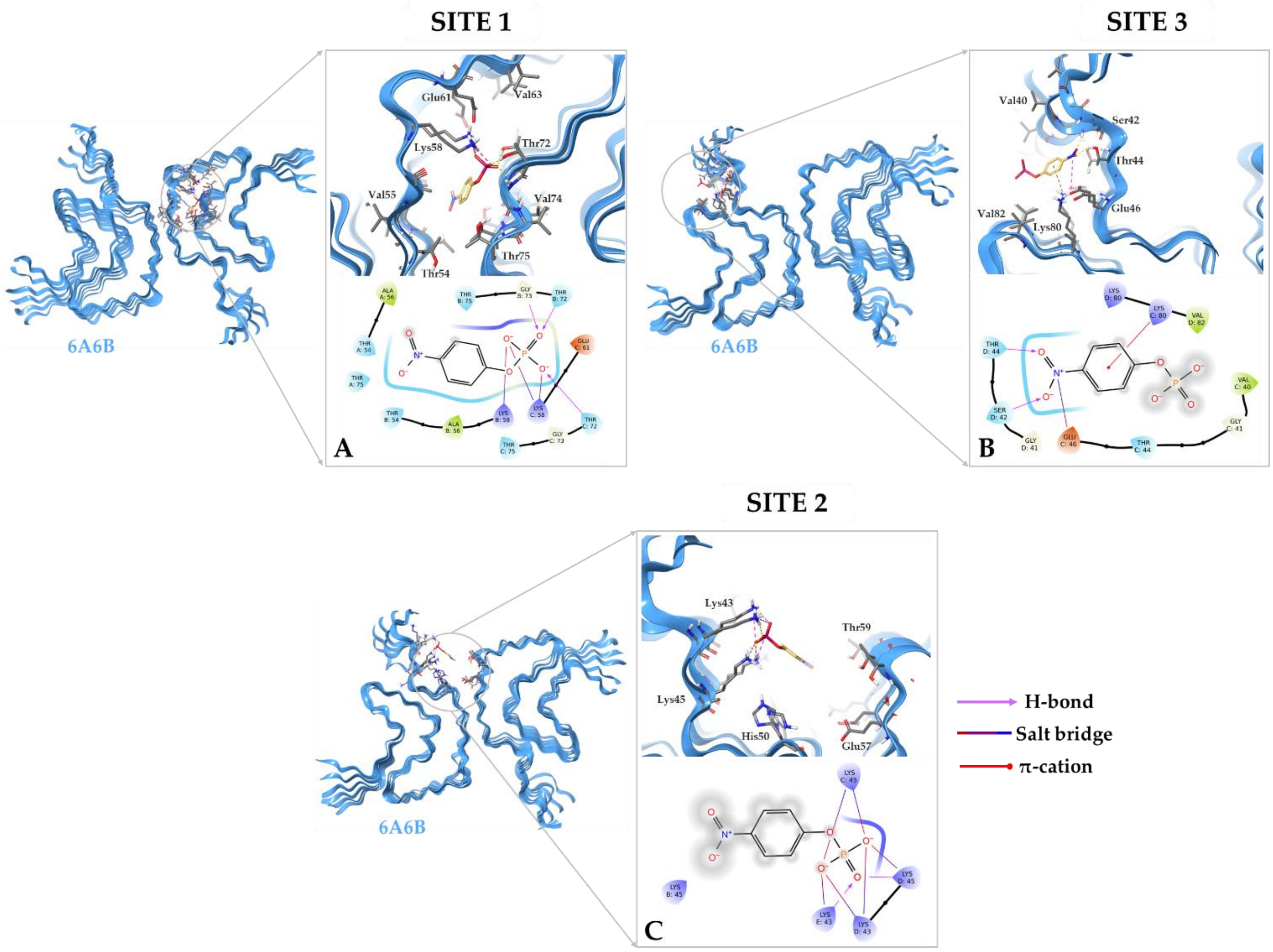

Figure S1. Molecular docking poses of pNPP when placed at the three identified binding sites (A, site 1; B, site 2; C, site 3) on αSyn fibrils (PDB: 6A6B). The corresponding 2D and 3D interaction diagrams were generated using Maestro Schrödinger.

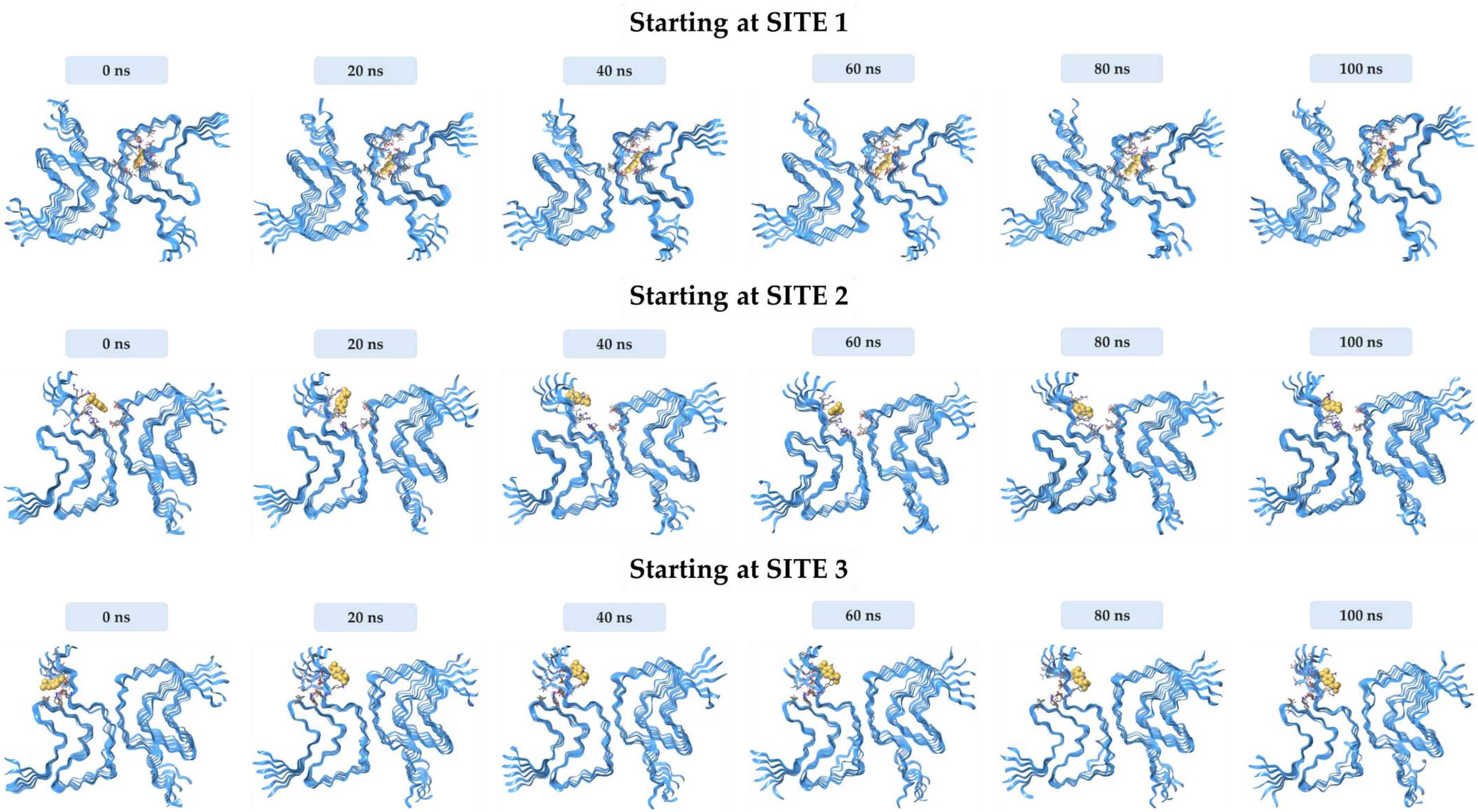

Figure S2. Snapshots of the pNPP interaction site, after docking (time zero) at one of the identified sites on 6A6B as indicated, at different time points during 100 ns of MD simulation.

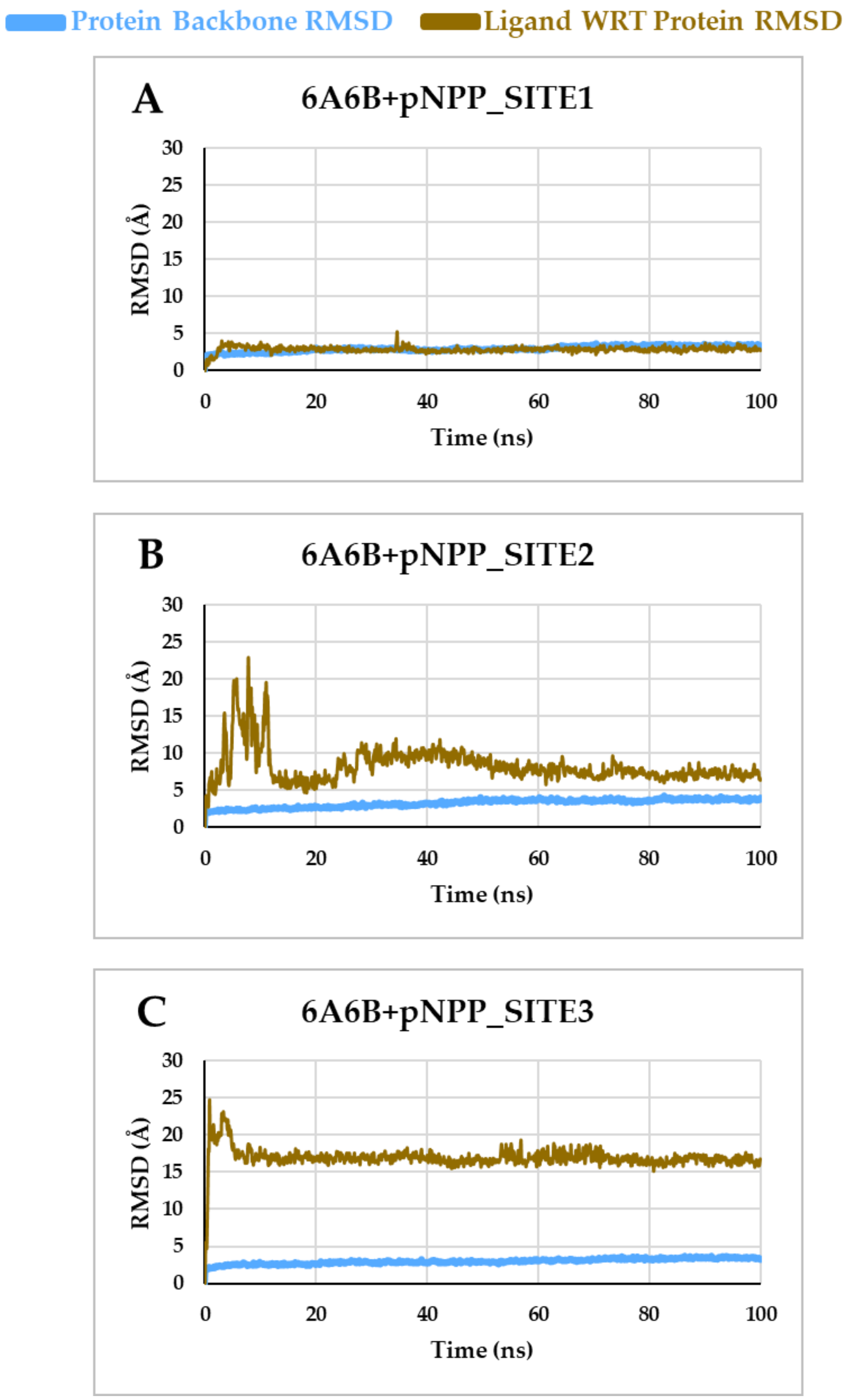

Figure S3. The backbone root mean square deviation (RMSD) analysis of αSyn fibrils (PDB: 6A6B) during MD simulation of 100 ns with pNPP initially docked at the three identified binding sites as indicated.

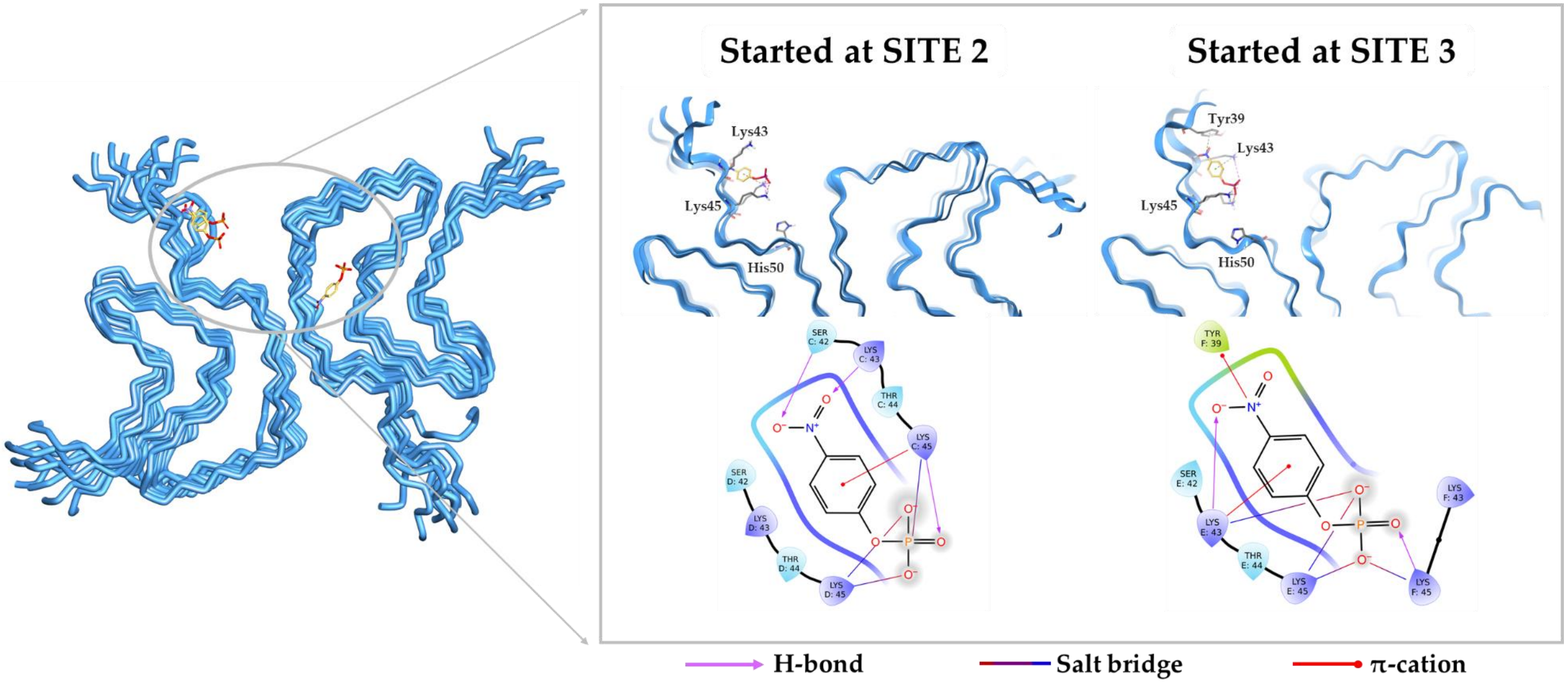

Figure S4. Representative snapshots from the end of 100 ns simulations of pNPP and the 6A6B structure showing resulting interaction profiles in Site 2. The corresponding 2D and 3D interaction diagrams were generated using Maestro Schrödinger.

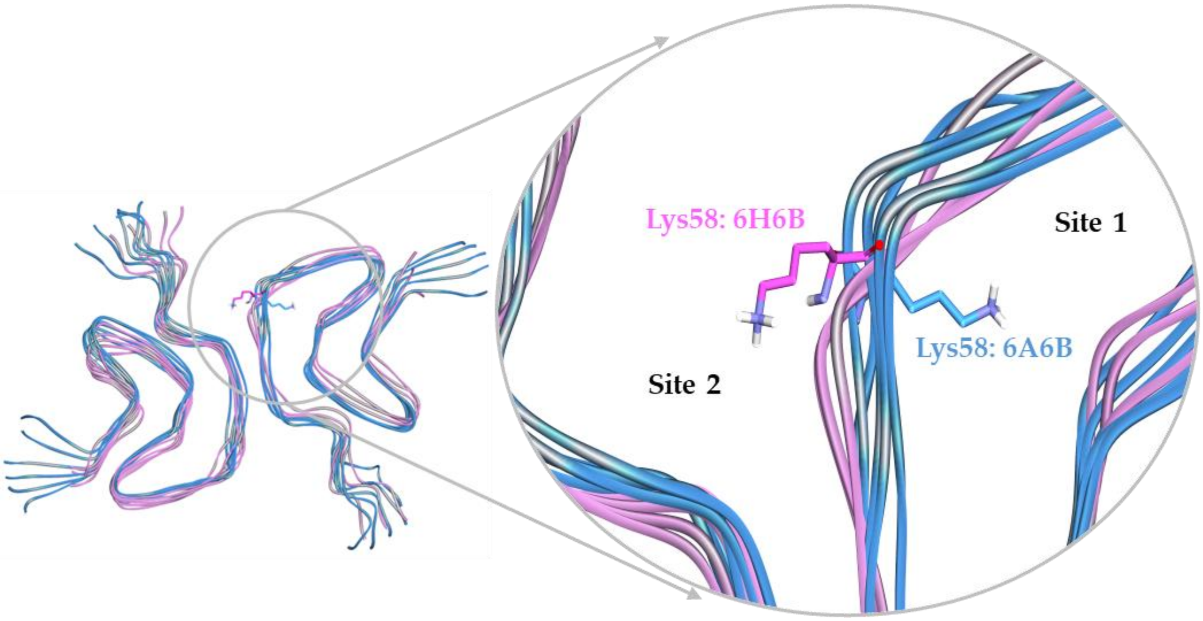

Figure S5. Superimposition of 6H6B and 6A6B fibril structures highlight differences in residue Lys58 positioning in relation to pNPP docked in Site 1. Left: Backbone alignment of the two fibril structures (6H6B in pink, 6A6B in blue) with a zoomed-in view of the binding region. Right: Close-up of Sites 1 and 2 showing the relative orientations of Lys58. In 6A6B, Lys58 is oriented towards Site 1, potentially stabilizing pNPP and preventing its migration to Site 2, as observed in 6H6B.
